# Transformation of Polycythemia Vera to Pure Erythroid Leukemia

**DOI:** 10.7759/cureus.16168

**Published:** 2021-07-04

**Authors:** Louisa Liu, Neil Dudheker, Lhara M Sumarriva Lezama, Sameer Shah, Maureen Nwaokoro, Vishal Ranpura

**Affiliations:** 1 Internal Medicine, University of California, Riverside, Riverside, USA; 2 Hematology and Oncology, University of California San Diego, San Diego, USA; 3 Pathology, Kaiser Permanente, Los Angeles, USA; 4 Hematology and Oncology, University of California Riverside, Riverside, USA; 5 Internal Medicine, University of California Riverside, Riverside, USA; 6 Hematology and Oncology, Kaiser Permanente, Riverside, USA

**Keywords:** pure erythroid leukemia, hydroxyurea, polycythemia vera, leukemic transformation, chronic myeloproliferative neoplasms

## Abstract

Pure erythroid leukemia (PEL) is an aggressive and exceedingly rare form of acute leukemia characterized as a neoplastic proliferation of immature cells committed to the erythroid lineage. It has a poor overall median survival of two to three months. To our knowledge, there are currently only a handful of reports of PEL arising from polycythemia vera. Most reported cases have been associated with radiation therapy or chemotherapeutic alkylating agents. Here we report a rare occurrence of polycythemia vera treated with phlebotomy and hydroxyurea that underwent leukemic transformation to pure erythroid leukemia.

## Introduction

Polycythemia vera (PV) is a chronic myeloproliferative neoplasm (MPN) caused by the overproliferation of red blood cells by the bone marrow. Current therapeutic options for PV (e.g., low-dose aspirin, phlebotomy, hydroxyurea) aim at reducing vascular and thrombotic complications. PV also carries the risk of leukemic transformation as a rare and late event in the disease [1]. In our case, the leukemic transformation occurred in the form of pure erythroid leukemia (PEL).

PEL is an aggressive and exceedingly rare form of acute leukemia. In 2016, World Health Organization (WHO) classified it as a subtype of acute myeloid leukemia and the only type of acute erythroid leukemia [2]. Currently, it accounts for less than 1% of all cases of acute myeloid leukemia (AML). PEL is characterized as a neoplastic proliferation of maturation-arrested erythroid precursors. More specifically, it is comprised of >80% immature erythroid precursors, of which 30% are proerythroblasts [3]. Compared to other cases of AML with erythroid hyperplasia, PEL is a unique entity that features a complex karyotype and is morphologically and immunophenotypically distinct [4]. The prognosis of patients with PEL is dismal, with overall median survival of two to three months.

To our knowledge, there are currently only a handful of reports of PEL arising from PV. Most reported cases have been associated with radiation therapy and chemotherapeutic alkylating agents [5-8]. Here we report a rare occurrence of polycythemia vera treated with phlebotomy and hydroxyurea that underwent leukemic transformation to pure erythroid leukemia. Unlike many previously reported cases of PEL arising from PV, our patient was neither treated with radiation therapy nor chemotherapy with alkylating agents.

## Case presentation

We present the case of a 67-year-old female with a history of janus kinase 2 (*JAK2) V617F-* positive polycythemia vera, managed for seven years with phlebotomy, aspirin, and hydroxyurea, presenting to the clinic with complaints of muscle aches, fatigue, loss of appetite, lightheadedness, and night sweats for two months. She was found to have developed petechiae on her upper extremities in addition to previous petechiae on her lower extremities. She was also noted to have a palpable spleen on physical exam. Complete blood count showed white blood cells (WBC) 7.9, Hgb 13.0, hematocrit (Hct) 41.8, platelets 63. She was advised to stop hydroxyurea and aspirin at the time.

The following month (May 2020), the patient returned to the clinic with a complaint of continued fatigue. In addition, she had noticed her urine becoming darker and her legs becoming more swollen. Blood workup was notable for leukocytosis (WBC 17.4), anemia [hemoglobin (Hgb) 11.8, Hct 37.6], and thrombocytopenia (18). Manual differential was notable for increased bands (22%), elevated lymphocytes (5.92), elevated monocytes (1.74), elevated metamyelocytes (0.52), elevated myelocytes (1.74), elevated promyelocytes (0.70). She was sent to the emergency department (ED) for transfusion. In the ED, she had an abdominal ultrasound performed, which showed hepatosplenomegaly with spleen measuring 26 cm in length and liver measuring 23.48 cm in craniocaudal diameter.

**Figure 1:**
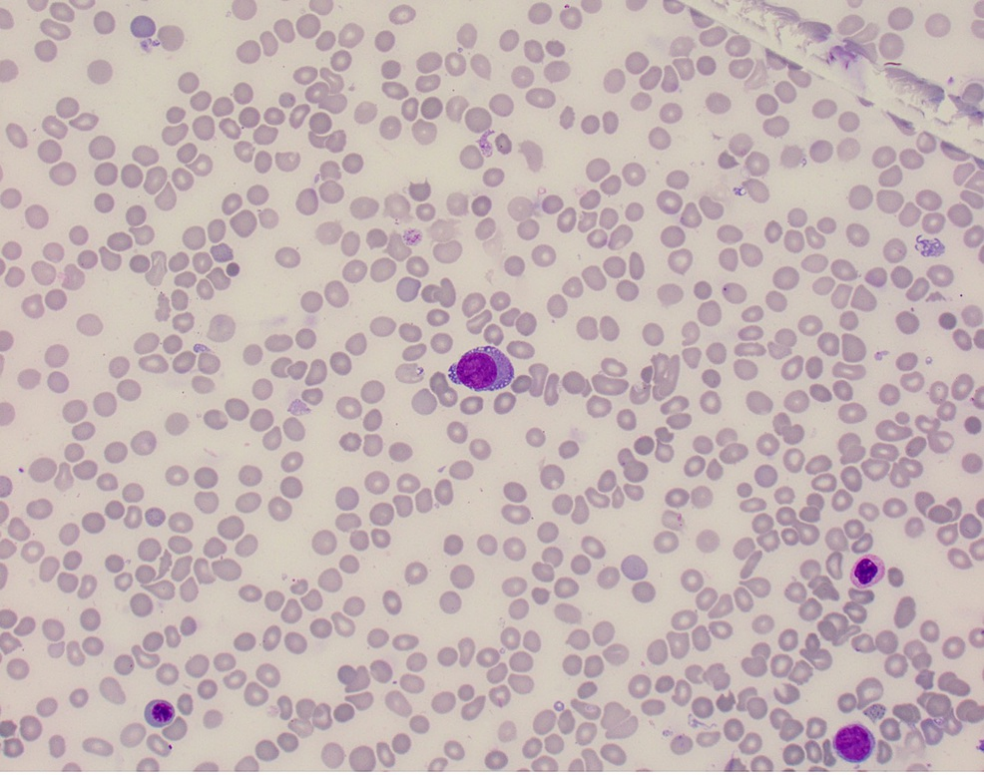
Bone marrow aspirate at 40X 40X magnification view of patient's bone marrow aspirate showing an erythroblast with large and round nuclei, and deep blue vacuolated cytoplasm.

**Figure 2:**
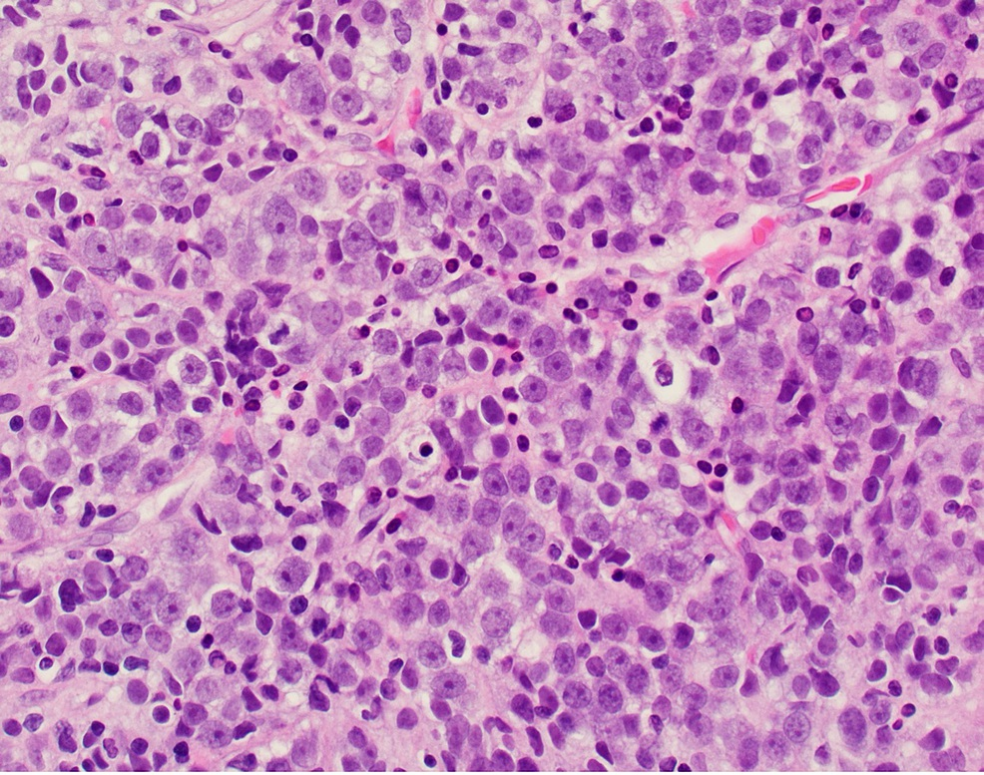
Hematoxylin & eosin at 40X 40X magnification view of hematoxylin & eosin (H&E) stained section showing sheets of erythroblasts, comprising ~90% of marrow cellularity.

**Figure 3:**
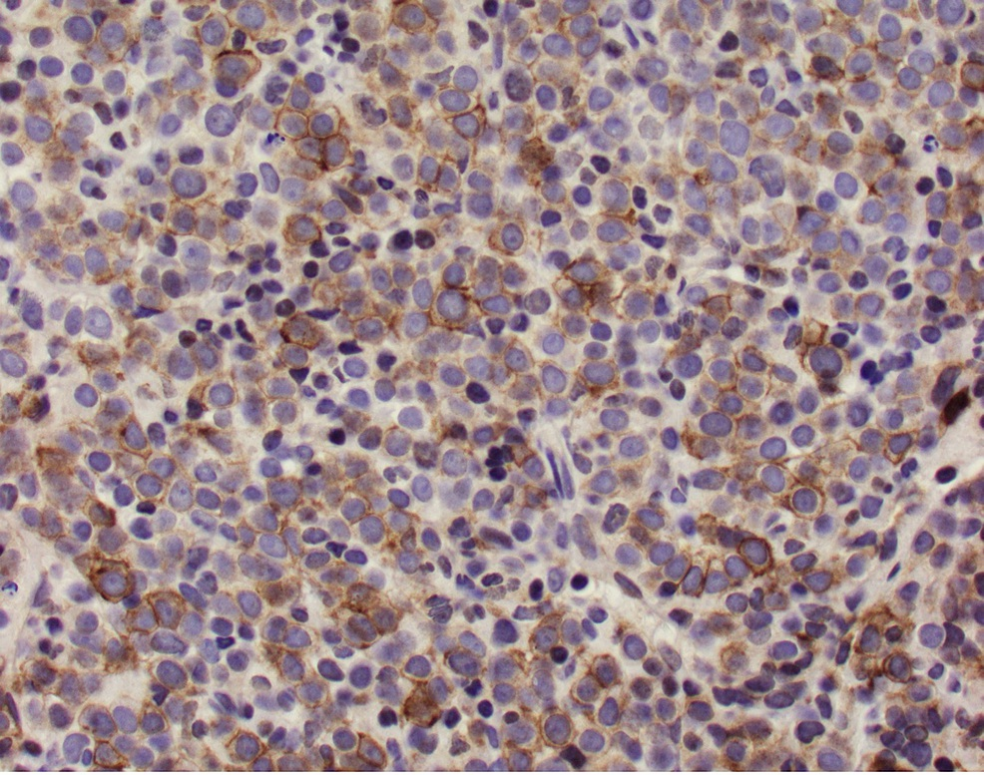
CD117 at 40X 40X magnification view of CD117 immunohistochemical stain highlighting the erythroid precursors.

**Figure 4:**
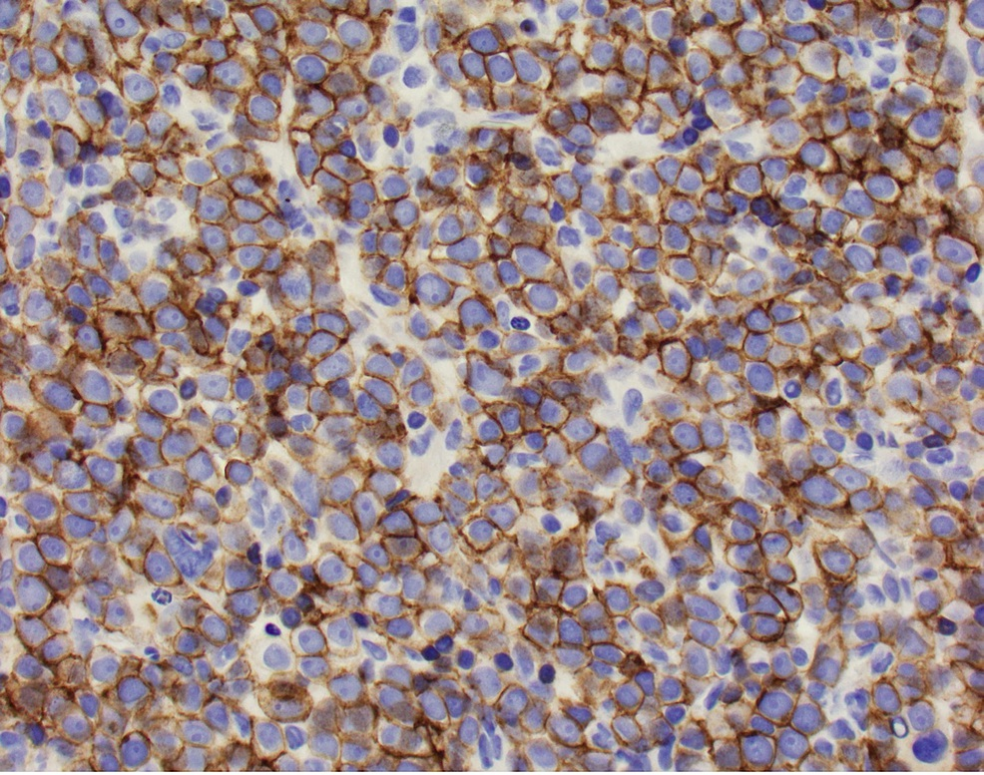
E-cadherin at 40X 40X magnification view of E-cadherin immunohistochemical stain highlighting the erythroid precursors.

The patient underwent a bone marrow biopsy due to concern for myelofibrosis. Bone marrow aspirate showed numerous blasts displaying a high N:C ratio, large nuclei, fine chromatin, prominent eosinophilic nucleoli, and deep blue cytoplasm, including some blasts showing vacuolated cytoplasm (Figure 1). The bone marrow core biopsy showed sheets of blasts, comprising ~90% of marrow cellularity (Figure 2). Immunohistochemical studies showed that the blasts expressed CD34 (partial), CD117 (variable), and E-cadherin; and lacked expression of terminal deoxynucleotidyl transferase (TdT), CD79a, CD3, myeloperoxidase (MPO), and factor 8 (Figure 3, Figure 4). These findings were consistent with pure erythroid leukemia. PEL diagnosis was further supported by finding of a complex karyotype on cytogenetic analysis: 46, XX, r(17) (p11.2q11.2), deletion (20) (q11.2q13.3), duplication (21) (q21q22), -22, +mar(20). Fluorescence in situ hybridization (FISH) AML panel was positive for an extra copy of the Runt-related transcription factor (RUNX) 1 (21q22) region, or trisomy 21 (49% of cells). 

Unfortunately, the patient was a poor candidate for aggressive chemotherapy and was started on non-intensive chemotherapy with azacytidine and venetoclax. She continued to spike fevers with worsening thrombocytopenia. After discussion with the patient and family regarding goals of care, the patient was transitioned to comfort-focused care and discharged on hospice. She passed away two weeks later.

## Discussion

Polycythemia vera is a chronic myeloproliferative neoplasm (MPN) characterized not only by erythrocytosis but also by overproduction of the myeloid and megakaryocytic components in the bone marrow. The most prevalent complications in patients with PV are vascular and thromboembolic events, such as strokes, acute coronary syndromes, deep vein thromboses of the extremities, and pulmonary emboli. Given the high mortality associated with thrombotic events in patients with PV, the goal of current therapies is to reduce the risk of thrombosis. Initial treatment depends on each patient’s thrombotic risk. Patients are stratified into “high-risk” or “low-risk” categories according to their age and history of thrombosis [9]. Patients under 60 years of age who have no prior history of thrombosis are considered low-risk and are treated with low-dose aspirin and phlebotomy. Patients over 60 years of age and/or who have a prior history of thrombosis are considered high-risk for vascular complications and are treated with cytoreductive therapy like hydroxyurea or interferon-alpha (IFN-α). As the standard first-line therapy for PV, hydroxyurea is responsible for decreasing the production of all cell lines produced in the bone marrow and is useful in controlling erythrocytosis, thrombocytosis, and splenomegaly in patients. IFN-α, another agent that has been shown to have antiproliferative effects on hematopoietic precursor cells, can be used in place of hydroxyurea for myelosuppression [10]. Despite treatment, however, a small subset of patients suffers from a longer-term complication of PV: hematological transformations.

Currently, there are two recognized phases of PV. The first phase is an initial polycythemic phase characterized by an elevated hemoglobin level and increased red blood cells (RBC) mass. The later spent phase, also known as the post-polycythemia myelofibrosis phase, is characterized by ineffective hematopoiesis, extramedullary hematopoiesis, cytopenia, and bone marrow fibrosis. A rare event that can occur late in the progression of PV is the evolution to acute leukemia, also known as a myelodysplastic or blast phase. An international multicenter study estimates the cumulative incidences of leukemic transformation at 2.3% at 10 years after initial diagnosis of PV, 5.5% at 15 years, and 7.9% at 20 years [11]. This transformation occurs often in the form of AML, although a few cases of lymphoblastic transformation have also been reported [12, 13].

In our case, the patient underwent a transformation to a rare subtype of AML known as PEL. PEL represents a neoplastic proliferation of immature cells committed to the erythroid lineage. 2016 WHO classification scheme classifies PEL as the only subtype of acute erythroid leukemia [2]. It is defined as at least 80% of erythroid precursor marrow involvement without an increase in myeloblasts. The erythroid cells in PEL are arrested at various stages of maturation and consist of undifferentiated erythroid precursors known as proerythroblasts, which are identified in the bone marrow by their large size, central round nuclei, and deeply basophilic cytoplasm. PEL is clinically aggressive, resulting often in rapidly progressive deterioration and death, as in our case. 

PEL evolving from an MPN is exceedingly rare, with only a few cases reported to our knowledge. Between 1952 and 1962, five cases detailing the transformation of PV to PEL were documented in patients treated with radioactive phosphorus or alkylating agents [5-8]. Kreft et al. reported a case of PEL arising from essential thrombocythemia (ET) [13]. Hongmei Li et al. reported one patient with PEL that progressed from ET and three patients with PEL that progressed from long-standing PV treated with phlebotomy and hydroxyurea [14]. Although the true incidence of transformation from PV to PEL is unknown, it is worth noting such a small number of documented cases reporting the phenomenon, despite the fact that both disorders are of erythroid lineage. 

The mechanism underlying leukemic progression remains unclear and has been suggested to be multifactorial. Advanced age and leukocytosis are well-established risk factors in patients with PV for progression to myelofibrosis. The European Collaboration on Low-Dose Aspirin in Polycythemia (ECLAP) study revealed that the persistence of leukocytosis despite treatment with hydroxyurea may serve as a marker of disease aggressiveness, indicating a greater risk of disease evolution and decreased survival in patients with this risk factor [15]. Other factors associated with leukemic transformation include previous exposure to myelosuppressive therapy, abnormal karyotypes, and certain mutations in genes such as serine/arginine-rich splicing factor (SRSF)2 and isocitrate dehydrogenase (IDH)1/2.

Our case highlights one of only a few reported instances of leukemic transformation from PV to PEL, without the presence of radiation therapy or alkylating agents. However, there remains some confusion in pinpointing the pathogenesis of PEL in our patient. Some studies have accepted leukemic transformation as a late event in the natural progression of PV [16, 17]. Our patient’s duration of disease and long-term use of hydroxyurea does not allow for easy discrimination between the risk of hematologic transformation due to the natural evolution of the disease, and that due to long-term pharmacologic cytoreduction. We speculate whether hydroxyurea may have a leukemogenic risk in PV. The high rate of mortality associated with leukemic transformation indicates a currently unmet therapeutic need of patients with PV. Although hydroxyurea use has increased over the last two decades, relatively little effort has been made in the assessment of its mutagenic potential. The use of cytoreductive drugs other than hydroxyurea and interferon has been significantly associated with an increased risk of leukemia [18]. However, hydroxyurea, alone or in addition to phlebotomy, has never been tested in any properly controlled trials with adequate statistical power. Moreover, clinical experience indicates that a substantial subset of patients remain on hydroxyurea therapy despite lack of response and intolerance. Hydroxyurea resistance has been found to increase the risk of death and transformation to myelofibrosis [19]. These findings denote a significant medical need for many patients with PV currently or previously treated with hydroxyurea. Accordingly, a group of experts convened by the European LeukemiaNet (ELN) recently developed a set of standardized criteria to monitor clinicohematologic response to hydroxyurea treatment in PV. Their unified definition of intolerance and resistance to hydroxyurea has been recommended to help clinicians decide on discontinuation of the drug [20]. It may be worth exploring whether phlebotomy alone, without the addition of hydroxyurea, should be used to treat those identified to have hydroxyurea-resistant PV.

Perhaps there may also be a role for alternative therapies such as janus kinase (JAK)2 inhibitors as a second-line treatment option for PV. Currently, second-line treatments for patients with PV at high risk for vascular complications include busulfan and radioactive phosphorus. However, studies observed that these agents have been associated with an increased risk of progression of the disease to AML or myelodysplastic syndromes [21]. In the randomized study of efficacy and safety in polycythemia vera with JAK inhibitor ruxolitinib versus best available care (RESPONSE) trial, ruxolitinib was compared with the best available therapy in patients with hydroxyurea-resistant or hydroxyurea-intolerant PV [22]. Ruxolitinib was found to be superior to standard therapy in achieving hematocrit control and reduction of symptom burden. In addition, patients treated with ruxolitinib had a lower thromboembolic event rate compared to those treated with the best available therapy. The adoption of JAK2 inhibitors as a next-line therapy for patients deemed to be hydroxyurea-resistant or intolerant will need to be investigated in future studies of larger size.

## Conclusions

Leukemic transformation to PEL represents a rare and fatal complication of PV that can occur even in the absence of treatment with known leukemogenic agents. As first-line therapy for PV, hydroxyurea use has been increasing over the last twenty years. However, the currently unmet therapeutic need and possible harm of patients with intolerance or resistance to hydroxyurea require that we investigate further into the mutagenic potential of hydroxyurea. This case also reveals another challenge, which will be the identification of patients who are at high risk of leukemic transformation. Going forward, it is anticipated that with advancements in genetic profiling, we will soon be able to identify the genetic mutations that lead to disease progression and transformation in MPNs, which will certainly be key to improving the classification, prognostication, and treatment of patients with PV.
